# Changes in the concentrations of creatinine, cystatin C and NGAL in patients with acute paraquat self-poisoning

**DOI:** 10.1016/j.toxlet.2011.01.024

**Published:** 2011-04-10

**Authors:** Darren M. Roberts, Martin F. Wilks, Michael S. Roberts, Ramasamyiyer Swaminathan, Fahim Mohamed, Andrew H. Dawson, Nick A. Buckley

**Affiliations:** a South Asian Clinical Toxicology Research Collaboration, University of Peradeniya, Peradeniya, Sri Lanka; b Department of Clinical Pharmacology and Toxicology, St. Vincent's Hospital, Darlinghurst, NSW, Australia; c Swiss Centre for Applied Human Toxicology, University of Basel, Basel, Switzerland; d Therapeutics Research Unit, University of Queensland, Brisbane, Australia; e School of Pharmacy & Medical Sciences, University of South Australia, Adelaide, Australia; f Department of Chemical Pathology, St. Thomas’ Hospital, London, UK; g Professorial Medicine Unit, POWH Clinical School, University of New South Wales, Randwick, NSW, Australia; h New South Wales Poisons Information Centre, The Children's Hospital, Westmead, NSW, Australia

**Keywords:** Paraquat, Prognosis, Kidney injury, Human, Creatinine, Biomarker, Cystatin C, NGAL

## Abstract

An increase in creatinine >3 μmol/L/h has been suggested to predict death in patients with paraquat self-poisoning and the value of other plasma biomarkers of acute kidney injury has not been assessed. The aim of this study was to validate the predictive value of serial creatinine concentrations and to study the utility of cystatin C and neutrophil gelatinase-associated lipocalin (NGAL) as predictors of outcome in patients with acute paraquat poisoning. The rate of change of creatinine (dCr/d*t*) and cystatin C (dCyC/d*t*) concentrations were compared between survivors and deaths. Receiver-operating characteristic (ROC) curves were constructed to determine the best threshold for predicting death. Paraquat was detected in 20 patients and 7 of these died between 18 h and 20 days post-ingestion. The dCr/d*t* ROC curve had an area of 0.93 and the cut-off was >4.3 μmol/L/h (sensitivity 100%, specificity 85%, likelihood ratio 7). The dCyC/d*t* ROC curve had an area of 0.97 and the cutoff was >0.009 mg/L/h (sensitivity 100%, specificity 91%, likelihood ratio 11). NGAL did not separate survivors from deaths. Death due to acute paraquat poisoning is associated with changes in creatinine and cystatin concentrations. Further validation of these measurements is needed before they can be adopted in guiding intensive treatments.

## 1 Introduction

Paraquat (1,1′-dimethyl-4,4′-bipyridinium) dichloride is a non-selective contact herbicide widely used in many countries since the 1960s. It is an important cause of fatal self-poisoning in some countries, particularly in South-East Asia (Gunnell et al., 2007). The outcome of paraquat poisoning is variable but in large cohort studies typically between 40 and 60% of cases die, most within 24–72 h from multi-organ failure (Dawson et al., 2010; Gil et al., 2008; Senarathna et al., 2009). However, patients with smaller exposures may die over the following weeks from respiratory failure secondary to progressive pulmonary fibrosis. Better prognostic indicators to identify this group would be very useful as ongoing interventions are most likely to be beneficial for this group with delayed toxicity. Paraquat produces free radicals which induce cellular toxicity (Eddleston et al., 2003). Many treatments have been proposed and trialled, including extracorporeal elimination, immunosuppressants and antioxidants, but the mortality remains high even in centres using all these treatments (Gil et al., 2008) (and JL Lin, unpublished observation 2010).

A very strong predictor of death in large cohort studies is the volume of paraquat consumed (Wilks et al., 2008, 2011), but estimates of this are often unreliable in individual patients. The concentration of paraquat in blood or urine can be used as a surrogate for ingested dose to predict survival or death using a nomogram. These have a positive predictive value for death of 92–96% (Senarathna et al., 2009). Unfortunately paraquat assays are not widely available, particularly in the developing world, and the time of ingestion may be unknown, so alternative biomarkers are required which should ideally be able to be interpreted independent of the time of exposure.

A range of alternative clinical and biochemical investigations for prognosis following acute paraquat poisoning have been assessed, but inadequately validated (Eddleston et al., 2003). For example, acute kidney injury is a prominent manifestation of acute paraquat poisoning which has prompted research into renal biomarkers (Gil et al., 2009; Ragoucy-Sengler and Pileire, 1996). One small study (*n* = 18) suggested that an increase in creatinine of >3 μmol/L/h (dCr/d*t*) predicts death (Ragoucy-Sengler and Pileire, 1996). The rise in creatinine is probably due to progressive renal impairment and a direct reflection of organ toxicity (Pond et al., 1993). However, paraquat interferes with some creatinine assays that utilise the Jaffe (picric-acid) method (Aitken et al., 1994; Fairshter et al., 1986; Price et al., 1995; Webb and Davies, 1981). Therefore, the increase in creatinine may reflect both exposure and toxicity. The apparent creatinine concentration increases with increasing paraquat concentrations (Aitken et al., 1994; Fairshter et al., 1986; Price et al., 1995; Webb and Davies, 1981), although minimally with concentrations less than 10 mg/L, in contrast to concentrations greater than 100 mg/L where interference is marked (Fairshter et al., 1986; Webb and Davies, 1981). In clinical practice the majority of paraquat concentrations are less than 100 mg/L (Senarathna et al., 2009).

The predictive value of other plasma biomarkers of acute kidney injury, such as cystatin C or neutrophil gelatinase-associated lipocalin (NGAL), have not been assessed in acute paraquat poisoning. A single study noted that urinary NGAL correlated with changes in creatinine concentration in patients with acute kidney injury (Gil et al., 2009).

The objective of this study was to further explore the utility of serial creatinine concentrations for predicting death and to examine the utility of plasma cystatin C and NGAL as alternative predictive biomarkers.

## 2 Materials and methods

This study was approved by Human Research Ethics Committees in Australia, Sri Lanka and UK. We prospectively identified all patients with acute paraquat exposure presenting to Anuradhapura and Polonnaruwa Hospitals in Sri Lanka. These are regional referral hospitals that provide 24-h medical and nursing care to patients in dedicated medical wards. Patients were directly admitted to a medical ward or via transfer from a remote hospital where they were medically assessed.

Every patient presenting to these study hospitals with a history of an acute paraquat exposure was reviewed by on-site study doctors. Following an initial clinical assessment and resuscitation, the history of exposure (including co-ingestants) was obtained on presentation for each patient. All patients received supportive care, including intravenous fluids and ventilatory and haemodynamic support as required; oxygen supplementation is withheld in patients with paraquat poisoning unless treatment is palliative and the patient is hypoxic. Patients were followed by dedicated study doctors until discharge or death. Follow up visits to the patient's home were attempted approximately 6 months after discharge to confirm survival.

Written informed consent was provided by 26 patients between 23rd April 2005 and 3rd September 2006 for the collection of additional blood samples. Blood samples were obtained at least 4 h post-ingestion (well after the peak plasma concentration), immediately centrifuged and plasma was taken off and stored at −23 °C until analysis. Samples were shipped to the UK to quantify the concentration of paraquat, creatinine and cystatin C. Available duplicate samples were shipped to Australia to quantify the concentration of NGAL.

Paraquat and creatinine analyses were conducted by Syngenta CTL (Alderley Park, Macclesfield, Cheshire, UK) in October 2006. The paraquat concentration was measured using HPLC, LC–MS–MS, and LC fluorescence (Blake et al., 2002). The creatinine concentration was measured utilising the modified Jaffe (picric-acid) method according to product guidelines (Labmedics, UK).

The cystatin C concentration was measured by Chemical Pathology, St. Thomas’ Hospital, London, UK in April 2007. This utilised a particle-enhanced nephelometric assay on a Dade Behring BNII nephlometer (Milton Keynes, UK) with antisera and calibrators supplied by Dade Behring (Bandaranayake et al., 2007).

NGAL concentration was measured by Therapeutics Research Centre, University of Queensland, Brisbane, Australia in October 2009. These assays were conducted using the Triage^®^ NGAL Test, a point-of-care fluorescence immunoassay using the Triage Meter according to product guidelines.

## 3 Calculation

Median values and inter-quartile ranges were determined for each renal biomarker and compared non-parametrically. The rate of change of creatinine and cystatin C concentrations in serial samples were determined and compared between survivors and deaths. Receiver-operating characteristic (ROC) curves were constructed to determine the best threshold (as determined by Youden's index (Youden, 1950)) for the rate of change of creatinine (dCr/d*t*) and cystatin C (dCyC/d*t*) concentrations for predicting death, including likelihood ratios, sensitivities and specificities. Sensitivity is the proportion of all deaths that were predicted to die by the test (cut-off), specificity is the proportion of survivors predicted to survive by the test. All analyses were conducted using GraphPad Prism version 4.03 for Windows, GraphPad Software, San Diego, USA, www.graphpad.com and *P* < 0.05 was considered statistically significant. Prediction of outcome on the basis of the admission paraquat concentration was determined according to Senarathna et al. (2009).

## 4 Results

### 4.1 Clinical outcomes

Paraquat exposure was confirmed in 20 patients who were eligible for inclusion; the other 6 patients were excluded. 14 of the 16 patients who were discharged alive were followed up in the community and three of these patients subsequently died. Altogether, seven patients died at 18 h, 48 h, 65 h, 11 days, 12 days, 15 days and 20 days after exposure. On the basis of the admission paraquat concentration, all actual deaths were predicted to die according to the Proudfoot nomogram (Eddleston et al., 2003). A total of 86 blood samples from different time points were assayed, although in some cases the volume was too small for every test to be conducted.

### 4.2 Changes in biomarkers of acute kidney injury post-admission

**Fig. 1:**
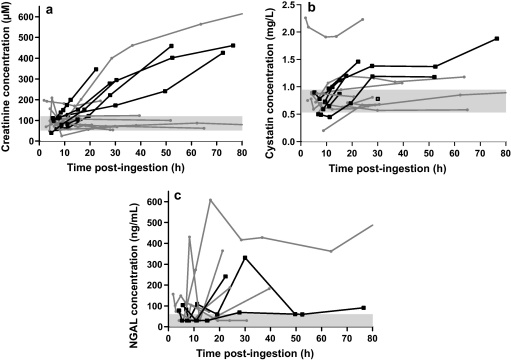
(a) Serial plasma creatinine concentrations in patients with acute paraquat poisoning, relative to outcome. (b) Serial plasma cystatin C concentrations in patients with acute paraquat poisoning, relative to outcome. (c) Serial plasma NGAL concentrations in patients with acute paraquat poisoning, relative to outcome. Values less than the reference range (<60 ng/mL) are plotted at 30 ng/mL for illustrative purposes. () Reference range; () death; () alive.

Serial concentrations of creatinine and cystatin C for individuals are shown in Fig. 1a and b, respectively. In the case of creatinine and cystatin C, increasing concentrations during the first 24–48 h were observed which were suitable for further analyses. Because biochemical data from patients who died were unavailable beyond 75 h post-ingestion, all subsequent analyses in surviving patients were limited to data obtained within the same period.

The plasma concentration of NGAL was measured in 14 patients and serial changes are shown in Fig. 1c. No relationship was observed that could be used to separate survivors from the four deaths captured in this study (which occurred 48 h, 65 h, 11 days and 12 days post-ingestion). Of these deaths, NGAL was not elevated in one patient while in the other three patients the highest concentration was 331 ng/mL and most were less than 100 ng/mL. The NGAL plasma concentration in one survivor was as high as 608 ng/mL.

### 4.3 Temporal trends in biomarkers of acute kidney injury

Where multiple samples were available in a single patient, in many cases the rates of increase in creatinine and cystatin C concentrations were approximately linear for the deaths (Fig. 1a and b) and the overall rate of change (estimated by linear regression of all samples) was used to construct ROC curves. The dCr/d*t* and dCyC/d*t* in survivors were also estimated using linear regression for direct comparison to data from survivors.

**Fig. 2:**
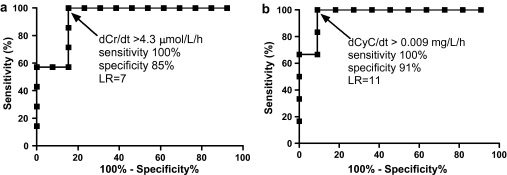
(a) Receiver-operator characteristic curve of dCr/d*t* for predicting death using linear regression of data within 75 h of poisoning. (b) Receiver-operator characteristic curve of dCyC/d*t* for predicting death using linear regression data within 75 h of poisoning.

Of the 13 survivors, only four were found to have a positive gradient for dCr/d*t* that was statistically different to zero (data not shown). The gradients were much higher for deaths [medians 9.0 μmol/L/h (IQR 5.3–14.8) for deaths and 0.3 μmol/L/h (IQR −0.3 to 3.3) for survivors; *P* = 0.002, Mann–Whitney test]. The ROC curve had an area of 0.93 (95% CI 0.83–1.04). The best dCr/d*t* cut-off was >4.3 μmol/L/h (sensitivity 100%, specificity 85% and likelihood ratio 7) (Fig. 2a).

Of the 11 survivors for which dCyC/d*t* results were available, only one trend line was found to have a positive gradient and to be statistically different to zero (data not shown). The gradients were again statistically greater for deaths [median 0.049 mg/L/h (IQR 0.017–0.074) for deaths and 0.004 mg/L/h (IQR −0.004 to 0.005) for survivors; *P* = 0.0022, Mann–Whitney test]. The dCyC/d*t* ROC curve had an area of 0.97 (95% CI 0.90–1.04) and the best cutoff was determined to be >0.009 mg/L/h (sensitivity 100%, specificity 91% and likelihood ratio 11) (Fig. 2b).

In one of these patients the dCr/d*t* and dCyC/d*t* exceeded values noted in deaths (Fig. 1a) and the creatinine concentration fulfill criteria for acute renal failure. This patient was not predicted to die according to the admission paraquat concentration. This patient survived to hospital discharge without receiving haemodialysis, but was lost to follow up so it is not known whether death occurred later. Excluding the two patients discharged alive but unable to be found at follow-up (creatinine data available for both patients but cystatin C data only available in one) improved the predictive value of creatinine but did not substantially alter the results of this analysis for cystatin C. Specifically, dCr/d*t* ROC AUC = 0.96 (95% CI 0.87–1.05); best cutoff >4.3 mg/L/h (sensitivity 100%, specificity 91% and likelihood ratio 11), and dCyC/d*t* ROC AUC = 0.97 (95% CI 0.89–1.05); best cutoff >0.009 mg/L/h (sensitivity 100%, specificity 90% and likelihood ratio 10.

**Fig. 3:**
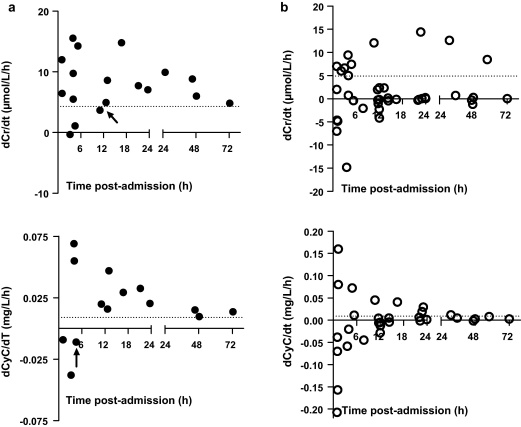
(a) Influence of the time of the second sample on the estimated rate of change of the concentration of creatinine (dCr/d*t*, upper graph) and cystatin C (dCyC/d*t*, lower graph) in patients who died. Arrow depicts the last value below the cut-off threshold determined by the ROC. (b) Influence of the time of the second sample on the estimated rate of change of the concentration of creatinine (dCr/d*t*, upper graph) and cystatin C (dCyC/d*t*, lower graph) in patients who survived.

However, as noted in Fig. 1a and b, the concentration of creatinine and cystatin C did not increase (or decrease) consistently in every patient. Therefore, dCr/d*t* and dCyC/d*t* values as determined by linear regression could vary depending on the time of sampling. To evaluate the minimum duration of sampling post-admission for assessing the dCr/d*t* or dCyC/d*t*, the rates of change from the time of admission to each subsequent blood sample for an individual patient were determined. Post-admission, on the basis of the available data, sampling after a minimum of approximately 12 h appeared necessary to predict death for dCr/d*t*, compared to approximately 6 h for dCyC/d*t* (Fig. 3a). However, the relationship was less precise for survivors (Fig. 3b), consistent with the test being sensitive for death but not specific (Fig. 3b).

### 4.4 Illustrative, data-rich cases

**Fig. 4:**
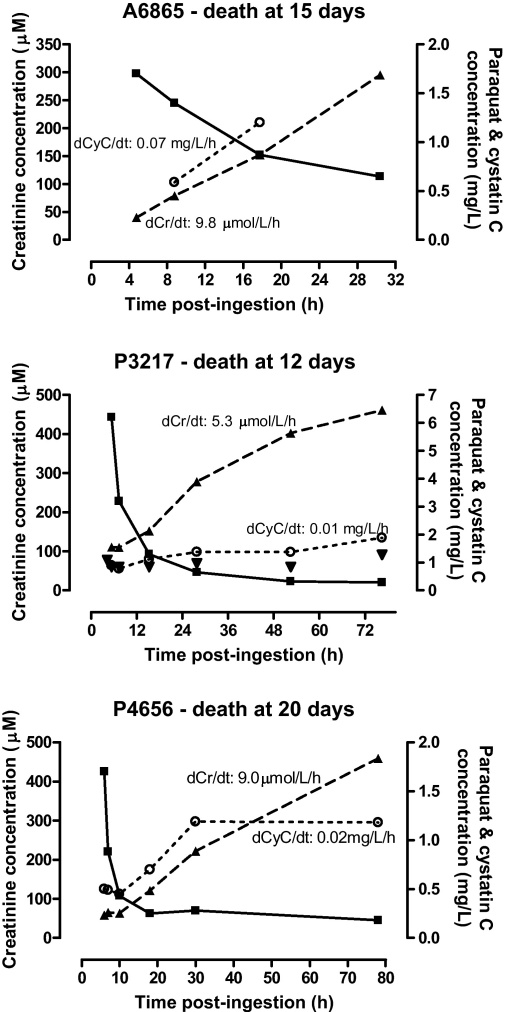
Serial changes in paraquat, creatinine and cystatin C concentrations for three patients with fatal outcome. The mean rates of change of creatinine and cystatin C concentrations in each individual are shown, (–■–) Paraquat; (--▴--) creatinine; (··○··) cystatin C.

Serial changes in creatinine and cystatin C plasma concentrations with time in three of the six deaths, relative to the concentration of paraquat, are shown in Fig. 4. The rates of change of creatinine and cystatin C are consistent with the results shown in Fig. 2a and b. Some patients had acute renal impairment on admission on the basis of creatinine and cystatin C concentrations, however these declined soon after admission which may be due to a component of hypovolaemia. In one patient (P4656, Fig. 4), the cystatin C concentration increased to a plateau while the concentration of creatinine continued to increase.

### 4.5 Potential for interference by paraquat with the creatinine assay

**Fig. 5:**
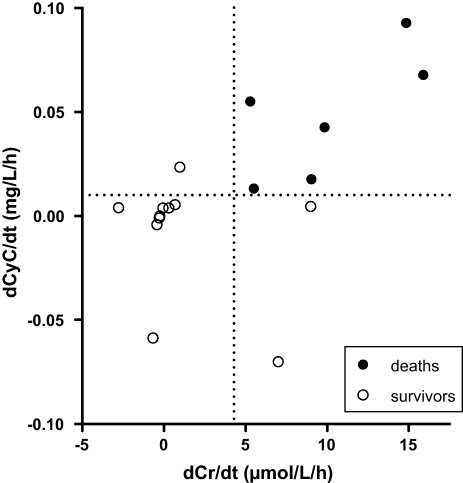
Relationship between dCr/d*t* and dCyC/d*t* in patients with acute paraquat poisoning.

Since the highest plasma paraquat concentration in this cohort was less than 10 mg/L, this was considered insufficient to interfere with the creatinine assay on the basis of laboratory data discussed previously. Therefore, no further analysis was conducted. As shown in Fig. 5, the rates of change in creatinine concentration correlated well with those of cystatin C. This is consistent with both measurements demonstrating progressive renal impairment.

## 5 Discussion

This small study confirms a previous report (Ragoucy-Sengler and Pileire, 1996) suggesting that the rate of change in creatinine concentration may be useful for predicting death after paraquat poisoning. Further, we demonstrated that the rise in cystatin C (but not NGAL) is also useful in predicting patients who may die. Due to the relatively low concentrations of paraquat observed in these patients it is unlikely that paraquat interfered with the creatinine assay However, even if there is direct interference this should not lead to rising concentrations of creatinine because the paraquat concentrations will be falling.

It is generally considered that paraquat poisoned patients most likely to benefit from antidotes or other treatments are those who will survive the first 48 h (Eddleston et al., 2003). As discussed previously, nomograms utilising the paraquat concentration can indicate the likelihood of death, but they do not differentiate between early and late deaths. Sawada et al. developed a nomogram using data from 30 patients which separated survivors, death by ‘circulatory failure’ and death by ‘respiratory failure’, but the time to death for each group was not stated (Sawada et al., 1988). Moreover, laboratories that measure paraquat concentrations are rare so alternative methods for risk stratification are required.

Paraquat induces acute tubular necrosis due to direct toxicity to the proximal tubule in particular, and to a lesser degree distal structures. Other factors influencing the development of acute kidney injury include hypoperfusion from hypovolaemia and/or hypotension and direct glomerular injury (Chan et al., 1998). Acute kidney injury and failure is diagnosed on the basis of changes in plasma creatinine concentration or urine output. In acute paraquat poisoning, creatinine peaks around five days post-ingestion and resolves within three weeks in survivors (Kim et al., 2009). In contrast to a previous study where acute renal failure was not noted in some patients who died (Gil et al., 2009), acute renal failure was noted in all deaths in our study (Fig. 3a); it also occurred in one survivor (Fig. 1a). Further, our study noted that dCr/d*t* predicts death where the best determinant was a rise >4.3 μmol/L/h (Fig. 2a), which is slightly higher than a rise >3 μmol/L/h in the previous study (Ragoucy-Sengler and Pileire, 1996). These rates of change in creatinine are likely to represent a loss of at least 70–80% of GFR (Waikar and Bonventre, 2009). At least 6–12 h need to lapse between samples to ensure that minor variation due to analytical error and rehydration does not obscure the change.

A large number of biomarkers of acute kidney injury have been described (Vaidya et al., 2008). Cystatin C is a 13 kD protein that is produced by all nucleated cells. It is freely filtered at the glomerulus, and in normally functioning renal tubules it is completely catabolised and reabsorbed. In response to acute kidney injury and/or inflammation there is an increase in concentration in both plasma and urine (Vaidya et al., 2008). The concentration of cystatin C is reported to increase prior to that of creatinine when there is a change in glomerular filtration rate, although this may vary between patient populations (Bagshaw and Bellomo, 2010). A significant increase in serum cystatin C concentration was observed 8 h following acute paraquat poisoning in rats; at 24 h an increase was also detected in bronchoalveolar fluid, however, it was suggested that this was due to leakage from the serum (Hantson et al., 2008a). A previous case report of acute paraquat poisoning also noted an increase in cystatin C and creatinine, similar to our findings (Hantson et al., 2008b). Our study is the first case series to evaluate the rate of change in cystatin C from acute paraquat poisoning. In survivors, cystatin C was noted to increase and plateau while creatinine continued to increase. This is likely to relate to a shorter half-life of cystatin C and therefore time to steady state compared to creatinine. In their patient (who died 92 h after admission) Hantson et al. reported a progressive increase in cystatin C concentrations until 70 h post-admission (Hantson et al., 2008b). However, this patient's treatment regimen included haemodialysis, which removes cystatin C from plasma (Mayeur et al., 2010), thereby complicating interpretation of this data.

NGAL is a 25 kD protein associated with neutrophils but is also produced by injured epithelial and renal tubular cells. It is freely filtered at the glomerulus. In response to acute kidney injury and/or inflammation there is an increase in concentration in both plasma and urine (Vaidya et al., 2008). Plasma NGAL appears to have diagnostic and prognostic value in acute kidney injury from various causes (Haase et al., 2009). However, in our study plasma NGAL did not correlate with survival (Fig. 1c). Urinary NGAL concentrations also appear inadequate as an early predictor of outcome with acute paraquat poisoning because the main increase was seen >48 h post-ingestion (Gil et al., 2009). Urinary kidney injury molecule-1 (KIM-1) may be a more sensitive marker of renal injury than creatinine, however, in a small study it did not appear to be useful for predicting death (Gil et al., 2009).

A limitation of this study is the small numbers of patients, which probably reflects the requirement for consent to obtain serial blood samples for the study. Patients with any significant ingestion of paraquat are generally told they have a grim prognosis by doctors who work in the hospitals where these patients are recruited (Roberts, unpublished observation). Therefore, it is not surprising many patients declined to participate to limit further discomfort (such as obtaining serial blood tests). Future studies offering new treatments are likely to be the best setting for recruiting sufficient numbers to further examine prognostic tests. Also, future studies should ensure that all patients are followed up a number of months post-discharge to ensure survival, compared to follow up of 90% of patients in this study.

Another limitation of this study is the delay in time to analysis. While the blood samples were stored frozen at −23 °C, it is possible that some degradation of NGAL during freezing may have occurred. This was reported in urine stored at −20 °C (Haase et al., 2009), but neither urine nor plasma samples stored at −80 °C (Haase et al., 2009; Pedersen et al., 2010).

The biomarkers evaluated here do not differentiate between early and late deaths and therefore cannot identify patients who are most likely to benefit from treatment.

## 6 Conclusion

The rate of increase in creatinine or cystatin C over the first 24 h may be useful for predicting outcomes in patients with acute paraquat poisoning. Prospective, larger cohort studies are required to confirm these findings and to more precisely determine the prognostic utility of these tests. Such studies should focus on the creatinine and cystatin C rise over the first 12–24 h. The notable short term random variation suggests measurements taken at shorter time intervals are more likely to be misleading. If properly validated, markers such as increases in creatinine or cystatin C may support clinical decisions on the first day regarding whether multiple complex treatments should be initiated in such patients, or if palliation is the priority. It may also be useful as part of the inclusion criteria for studies of new treatments.

## Conflict of interest statement

The authors affiliated with SACTRC (DMR, FM, AHD, NAB) have collaborated with employees of Syngenta which manufactures paraquat, and also Monsanto which manufactures other herbicides. These collaborations have led to research publications in the peer reviewed literature and no personal payments were received within the research funding. MW is a past employee of Syngenta.

## References

[bib0005] Aitken R.G., Northall H., York G.A. (1994). High serum concentrations of paraquat increase apparent creatinine concentrations. Ann. Clin. Biochem..

[bib0010] Bagshaw S.M., Bellomo R. (2010). Cystatin C in acute kidney injury. Curr. Opin. Crit. Care.

[bib0015] Bandaranayake N., Ankrah-Tetteh T., Wijeratne S., Swaminathan R. (2007). Intra-individual variation in creatinine and cystatin C. Clin. Chem. Lab. Med..

[bib0020] Blake D.K., Gallagher R.T., Woollen B.H. (2002). Improved methods for the analysis of paraquat in biological fluids. Chromatographia.

[bib0025] Chan B.S., Lazzaro V.A., Seale J.P., Duggin G.G. (1998). The renal excretory mechanisms and the role of organic cations in modulating the renal handling of paraquat. Pharmacol. Ther..

[bib0030] Dawson A.H., Eddleston M., Senarathna L., Mohamed F., Gawarammana I., Bowe S.J., Manuweera G., Buckley N.A. (2010). Acute human lethal toxicity of agricultural pesticides: a prospective cohort study. PLoS Med..

[bib0035] Eddleston M., Wilks M.F., Buckley N.A. (2003). Prospects for treatment of paraquat-induced lung fibrosis with immunosuppressive drugs and the need for better prediction of outcome: a systematic review. Q. J. Med..

[bib0040] Fairshter R.D., Miyada D.S., Ulich T.R., Tipper P. (1986). The effects of paraquat dichloride on clinical chemistry measurements. J. Anal. Toxicol..

[bib0045] Gil H.W., Kang M.S., Yang J.O., Lee E.Y., Hong S.Y. (2008). Association between plasma paraquat level and outcome of paraquat poisoning in 375 paraquat poisoning patients. Clin. Toxicol..

[bib0050] Gil H.W., Yang J.O., Lee E.Y., Hong S.Y. (2009). Clinical implication of urinary neutrophil gelatinase-associated lipocalin and kidney injury molecule-1 in patients with acute paraquat intoxication. Clin. Toxicol..

[bib0055] Gunnell D., Eddleston M., Phillips M.R., Konradsen F. (2007). The global distribution of fatal pesticide self-poisoning: systematic review. BMC Public Health.

[bib0060] Haase M., Bellomo R., Devarajan P., Schlattmann P., Haase-Fielitz A. (2009). Accuracy of neutrophil gelatinase-associated lipocalin (NGAL) in diagnosis and prognosis in acute kidney injury: a systematic review and meta-analysis. Am. J. Kidney Dis..

[bib0065] Hantson P., Bernard A., Hermans C. (2008). Kinetics and determinants of the changes of CC16, a lung secretory protein in a rat model of toxic lung injury. Clin. Toxicol. (Phila.).

[bib0070] Hantson P., Weynand B., Doyle I., Bernand A., Hermans C. (2008). Pneumoproteins as markers of paraquat lung injury: a clinical case. J. Forensic Leg. Med..

[bib0075] Kim S.J., Gil H.W., Yang J.O., Lee E.Y., Hong S.Y. (2008). The clinical features of acute kidney injury in patients with acute paraquat intoxication. Nephrol. Dial. Transplant..

[bib0080] Mayeur N., Rostaing L., Nogier M.B., Jaafar A., Cointault O., Kamar N., Conil J.M., Fourcade O., Lavayssiere L. (2010). Kinetics of plasmatic cytokines and cystatin C during and after hemodialysis in septic shock-related acute renal failure. Crit. Care.

[bib0085] Pedersen K.R., Ravn H.B., Hjortdal V.E., Norregaard R., Povlsen J.V. (2010). Neutrophil gelatinase-associated lipocalin (NGAL): validation of commercially available ELISA. Scand. J. Clin. Lab. Invest..

[bib0090] Pond S.M., Rivory L.P., Hampson E.C., Roberts M.S. (1993). Kinetics of toxic doses of paraquat and the effects of hemoperfusion in the dog. J. Toxicol. Clin. Toxicol..

[bib0095] Price L.A., Newman K.J., Clague A.E., Wilson P.R., Wenck D.J. (1995). Paraquat and diquat interference in the analysis of creatinine by the Jaffe reaction. Pathology.

[bib0100] Ragoucy-Sengler C., Pileire B. (1996). A biological index to predict patient outcome in paraquat poisoning. Hum. Exp. Toxicol..

[bib0105] Sawada Y., Nagai Y., Ueyama M., Yamamoto I. (1988). Probable toxicity of surface-active agent in commercial herbicide containing glyphosate. Lancet.

[bib0110] Senarathna L., Eddleston M., Wilks M.F., Woollen B.H., Tomenson J.A., Roberts D.M., Buckley N.A. (2009). Prediction of outcome after paraquat poisoning by measurement of the plasma paraquat concentration. Q. J. Med..

[bib0115] Vaidya V.S., Ferguson M.A., Bonventre J.V. (2008). Biomarkers of acute kidney injury. Annu. Rev. Pharmacol. Toxicol..

[bib0120] Waikar S.S., Bonventre J.V. (2009). Creatinine kinetics and the definition of acute kidney injury. J. Am. Soc. Nephrol..

[bib0125] Webb D.B., Davies C.G. (1981). Paraquat poisoning and kidney function tests. Lancet.

[bib0130] Wilks M.F., Fernando R., Ariyananda P.L., Eddleston M., Berry D.J., Tomenson J.A., Buckley N.A., Jayamanne S., Gunnell D., Dawson A. (2008). Improvement in survival after paraquat ingestion following introduction of a new formulation in Sri Lanka. PLoS Med..

[bib0145] Wilks M.F., Tomenson J.A., Fernando R., Ariyananda P.L., Berry D.J., Buckley N.A., Gawarammana I., Jayamanne S., Gunnell D., Dawson A. (2011). Formulation changes and time trends in outcome following paraquat ingestion in Sri Lanka. Clinical Toxicology.

[bib0140] Youden W.J. (1950). Index for rating diagnostic tests. Cancer.

